# Lower limb lengthening over an intramedullary nail: a long-term follow-up study of 28 cases

**DOI:** 10.1186/s10195-019-0538-y

**Published:** 2019-09-10

**Authors:** Pasquale Farsetti, Fernando De Maio, Vito Potenza, Kristian Efremov, Martina Marsiolo, Alessandro Caterini, Ernesto Ippolito

**Affiliations:** Department of Orthopaedic Surgery, University of Rome “Tor Vergata”, Rome, Italy

**Keywords:** Limb lengthening, External fixation, Intramedullary nail

## Abstract

**Background:**

Limb lengthening using an external fixator requires a long period of external fixation and may be associated with several complications such as axial deformity, fracture of the regenerated bone, and joint stiffness. With the goal of reducing the time of external fixation as well as some of these complications, we performed femoral or tibial lengthening over an intramedullary nail, according to Paley’s technique, in 28 patients, followed up after a mean period of 8 years.

**Materials and methods:**

Twenty-eight patients treated for lower limb discrepancy by limb lengthening over an intramedullary nail were reviewed from 5 to 11 years after healing of regenerated bone. There were 20 femurs and 8 tibiae, with average age at surgery of 14.2 years and average length inequality of 6.1 cm for femurs and 5.3 cm for tibiae.

**Results:**

The mean lengthening was 5.8 cm for femurs and 4.8 cm for tibiae. The mean period of radiographic consolidation of the regenerated bone was 6 months for femoral lengthening and 4.5 months for tibial lengthening. At follow-up, we observed 8 excellent results, 15 good results, 4 fair results, and 1 poor result, based on Paley’s evaluation criteria. The main complications were one deep infection, one nonunion of the distracted segment, one breakage of the distal fiche of the external fixator, and one breakage of both distal locking screws of the intramedullary nail.

**Discussion:**

We believe that limb lengthening over an intramedullary nail still represents a good method to treat limb length discrepancy because it reduces the time of external fixation, prevents axial deformities and fractures of regenerated bone, and allows early rehabilitation. The new intramedullary lengthening nails, which theoretically are the ideal device for treating limb length inequality, are still very expensive and need longer follow-up for definitive evaluation.

**Level of evidence:**

4.

## Introduction

Congenital or acquired limb length discrepancy is a relatively common pathologic condition, usually treated by limb lengthening with external fixation. This is a complex procedure with a high rate of complications, including vascular and nerve injuries, axial deviations, fractures of regenerated bone following external fixator removal, joint stiffness, and infection [1–7]. Paley et al. [8] described a new technique of femoral lengthening over an intramedullary nail 20 years ago, with the main goal of reducing the duration of external fixation for limb lengthening, since prolonged use of the external fixator is generally poorly tolerated by the patient. Moreover, this technique prevents axial deviations of the lengthened skeletal segment, fractures of the regenerated bone after removal of the external fixator, and joint stiffness. After that first description, several authors used this method for lengthening with satisfactory results [9–17]. Self-elongating intramedullary nails have recently been introduced (mechanical or motorized) to avoid the discomfort and problems of the external fixator, but their technology needs further improvements and their cost remains very high [18–27].

We report herein the long-term results obtained in 28 patients with lower limb discrepancy of the femur or tibia exceeding 4.5 cm, treated with Paley’s procedure.

## Materials and methods

**Fig. 1:**
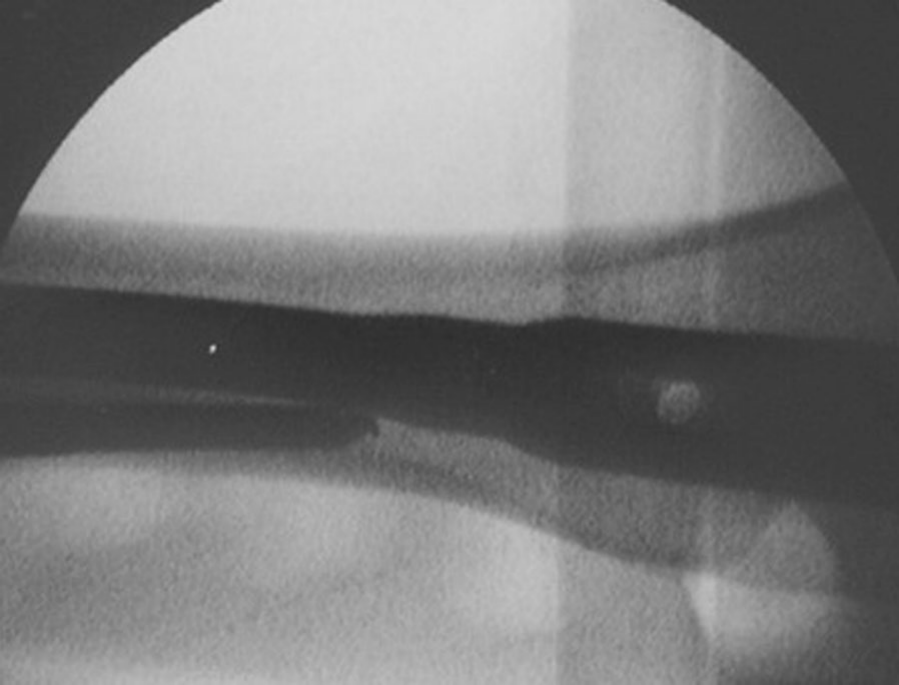
Intraoperative image intensifier spot of femur showing the relationship between the fiches and nail in lateral view

We retrospectively reviewed 28 patients affected by lower limb length discrepancy treated by lower limb lengthening over an intramedullary nail between 2004 and 2010. The limb length inequality was congenital in 16 patients and posttraumatic in 12. Twelve patients were male, and 16 were female. Lengthening of the femur was performed in 20 cases, and of the tibia in 8 cases. The average age of the patients at time of surgery was 14.2 years (range 10–21 years). The average length of lower limb discrepancy was 5.9 cm (range 4.6–9.2 cm). The average length of discrepancy was 6.1 cm (range 4.9–9.2 cm) for the femur and 5.3 cm (range 4.6–7.3 cm) for the tibia (Table 1). Lengthening of the femur was performed on the traction table. The first step consisted of introducing the intramedullary nail after reaming the medullary canal to 2 mm wider than the chosen nail. The second step consisted of inserting two or three external fixator fiches, 5.5 mm in diameter, proximally and distally to the planned osteotomy. The fiches were inserted posteriorly to the nail, if possible without any contact with the nail (Fig. 1). The third step consisted of corticotomy according to the De Bastiani technique [1], after partial withdrawal of the nail. The corticotomy was performed at the proximal or central part of the diaphysis, depending on the extent of the planned lengthening. The last step consisted of reinserting the nail, locked proximally with a screw, applying a uniplanar external fixator on the fiches, and starting the lengthening process. The initial lengthening was performed with distraction of 5 mm, verifying that there was no friction during the sliding of the bone segments on the nail. To perform tibial lengthening, we followed the same steps on a radiolucent surgical table, using a humeral intramedullary nail, 7 mm in diameter, and a circular Ilizarov-type fixator, stabilized to the bone only with crossed Kirschner wires.

**Table 1:** Demographics and results of patients

Patient no.	Age (years)	Sex	Side	Lengthening site	Limb length discrepancy (cm)	Lengthening achieved (cm)	Paley score
1	10	Female	Left	Femur	5.2	5.0	85
2	11	Female	Left	Femur	5.1	5.1	95
3	12	Male	Right	Femur	5.3	4.9	50
4	13	Male	Left	Femur	5.4	5.1	80
5	13	Female	Right	Femur	6.8	6.5	85
6	13	Male	Right	Femur	4.9	4.6	100
7	13	Female	Left	Femur	6.4	6.3	80
8	13	Male	Right	Femur	6.6	6.3	90
9	13	Male	Right	Femur	5.9	5.2	80
10	13	Male	Left	Femur	6.6	6.6	90
11	14	Female	Left	Femur	7.3	7.1	85
12	14	Female	Right	Femur	5.2	5.0	100
13	14	Female	Left	Femur	5.3	5.1	85
14	14	Female	Right	Femur	5.0	4.6	75
15	15	Female	Left	Femur	7.5	5.0	35
16	15	Male	Right	Femur	5.5	5.2	95
17	16	Male	Right	Femur	9.2	9	60
18	16	Male	Right	Femur	5.3	5.2	85
19	17	Female	Right	Femur	7.1	6.3	75
20	21	Female	Left	Femur	8.5	7.0	55
21	12	Female	Right	Tibia	4.7	4.1	85
22	13	Male	Right	Tibia	5.6	4.2	70
23	13	Male	Right	Tibia	5.1	5.2	95
24	14	Female	Left	Tibia	7.3	7.3	100
25	15	Male	Right	Tibia	5.2	5.2	100
26	15	Female	Left	Tibia	4.7	4.2	90
27	17	Female	Left	Tibia	5.1	4.4	80
28	18	Female	Left	Tibia	4.6	4.0	95

Paley score is considered excellent (95–100 points), good (75–94 points), fair (40–74 points), or poor (less than 40 points)

All surgical procedures were performed under general anesthesia, with standard antibiotic prophylaxis with cefazolin. Antithromboembolic prophylaxis was also administered. Distraction of 1 mm/day was started 1 week after surgery, until the planned length was reached. After hospital discharge, the patients continued distraction autonomously as instructed by the operating physician. Moreover, the patients were also instructed about how to handle the hygiene of the fiches or wires, and to immediately report any signs of infection (pin-tract infection, fever, pain, etc.). Patients were allowed partial weight-bearing with two crutches, and they started physical therapy between the fifth and seventh day postoperatively.

Patients were followed up by clinical and radiographic examinations every 2 weeks, to verify that lengthening of the bone segment was going as planned. Clinically, we verified the possible presence of persistent pain, fever, joint stiffness, skin disorders around the fiches, or signs of infection, and vascular or nerve dysfunction. Radiographically, we verified the progression of the lengthening without complications and the ossification of the regenerated bone. The speed of lengthening was slowed or stopped for a few days when persistent pain or vascular or nerve disorders were present or the ossification of the regenerated bone was delayed. When the planned length of the bone segment had been reached, the second surgical procedure was performed, consisting of removal of the external fixator and its fiches or K-wires and distal blockage of the nail with two screws. Following the second surgery, an intensive physical therapy program was started in order to regain full range of motion and muscle strength. Full weight-bearing was allowed when x-rays showed adequate bone bridges between the two lengthened skeletal segments.

At follow-up, all patients were evaluated clinically and radiographically, using the scoring system reported by Paley et al. [8], which is based on range of motion of the knee or ankle, amount of lengthening, gait, axial deviation, pain, and ability to perform everyday activities or to work. The scores were rated as excellent (95–100 points), good (75–94 points), fair (40–74 points), or poor (less than 40 points).

We performed statistical analysis of the study parameters. The Student *t*-test and Mann–Whitney *U*-test were used to evaluate differences between femoral and tibial lengthening in regards to lengthening achieved, duration of external fixation, and time for radiographic evidence of bone healing. Pearson’s product-moment correlation, Spearman’s rank correlation, and Kendall’s rank correlation were used to evaluate possible correlations between Paley score and patient age and lengthening achieved. All statistical analyses were performed using SigmaStat version 4.0 (SYSTAT) software. *p*-Value less than 0.05 was considered significant.

## Results

The mean duration of the surgical procedure was 3 h for both femoral and tibial lengthening (from 2.5 to 4.5 h). Mean intraoperative blood loss during the femoral procedures was 320 ml (from 150 to 700 ml), whereas in the tibial procedures there was no significant blood loss, because a thigh tourniquet was used.

The mean lengthening of the femurs was 5.8 cm (from 4.6 to 9 cm), whereas that of the tibiae was 4.8 cm (from 4 to 7.3 cm). This difference was statistically significant (*p* = 0.03).

The mean time of external fixation for femoral lengthening was 3.6 months (from 2 to 4.8 months), while for tibial lengthening it was 3 months (from 2 to 4.3 months). This difference was not statistically significant (*p* = 0.09).

The mean time for radiographic evidence of bone healing, following removal of the external fixator, was 6 months (from 4.3 to 8.5 months) for femoral lengthening but 4.5 months (from 3.8 to 7 months) for tibial lengthening. This difference was statistically significant (*p* = 0.02).

The intramedullary nail was removed in all cases, at least 8 months after radiographic consolidation of the lengthened bone segment.

**Fig. 2:**
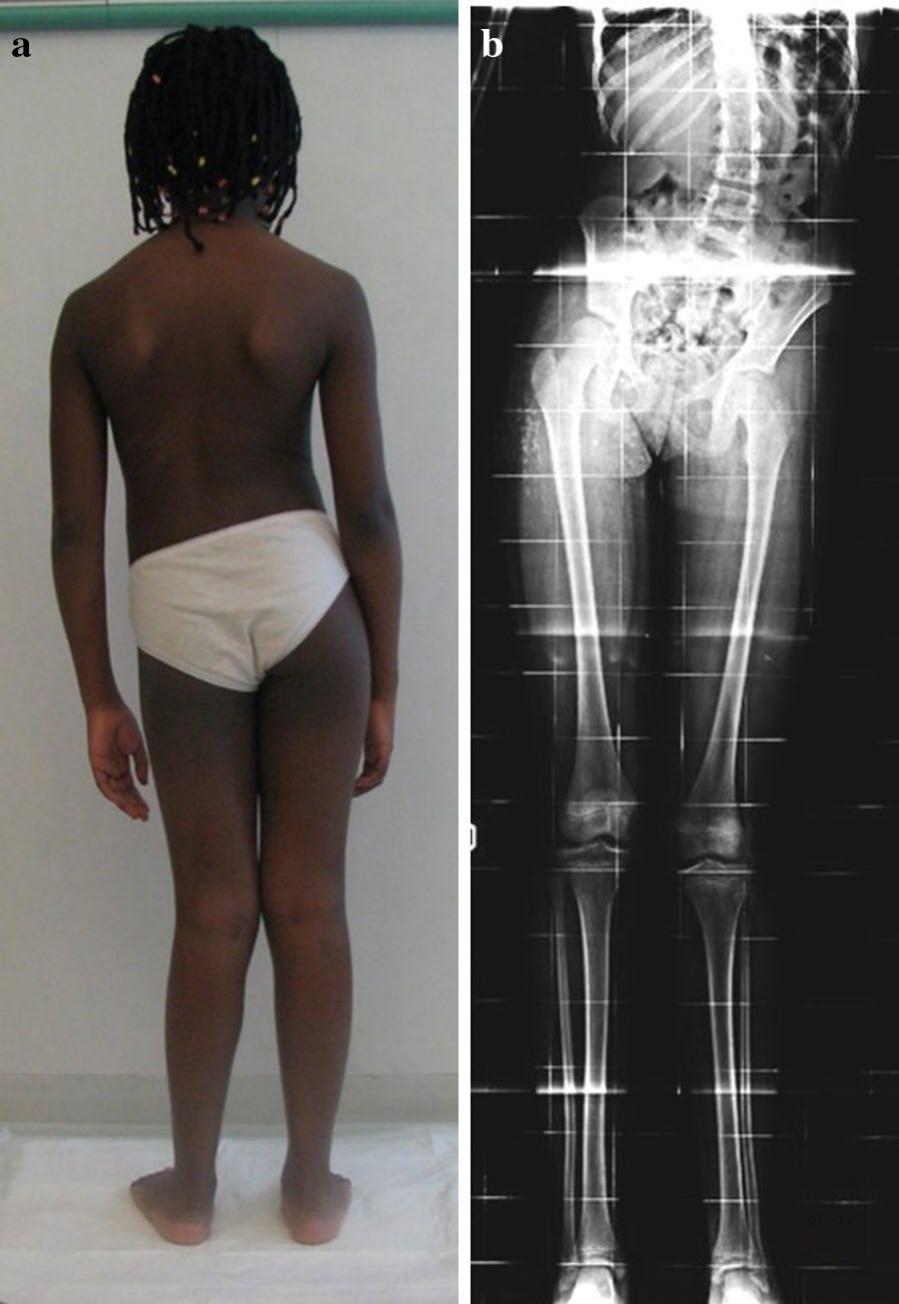
**a**, **b** Clinical and radiographic aspect of congenital lower limb length discrepancy in a 10-year-old patient. The length inequality was 5.2 cm for the left femur and 1.4 cm for the tibia

**Fig. 3:**
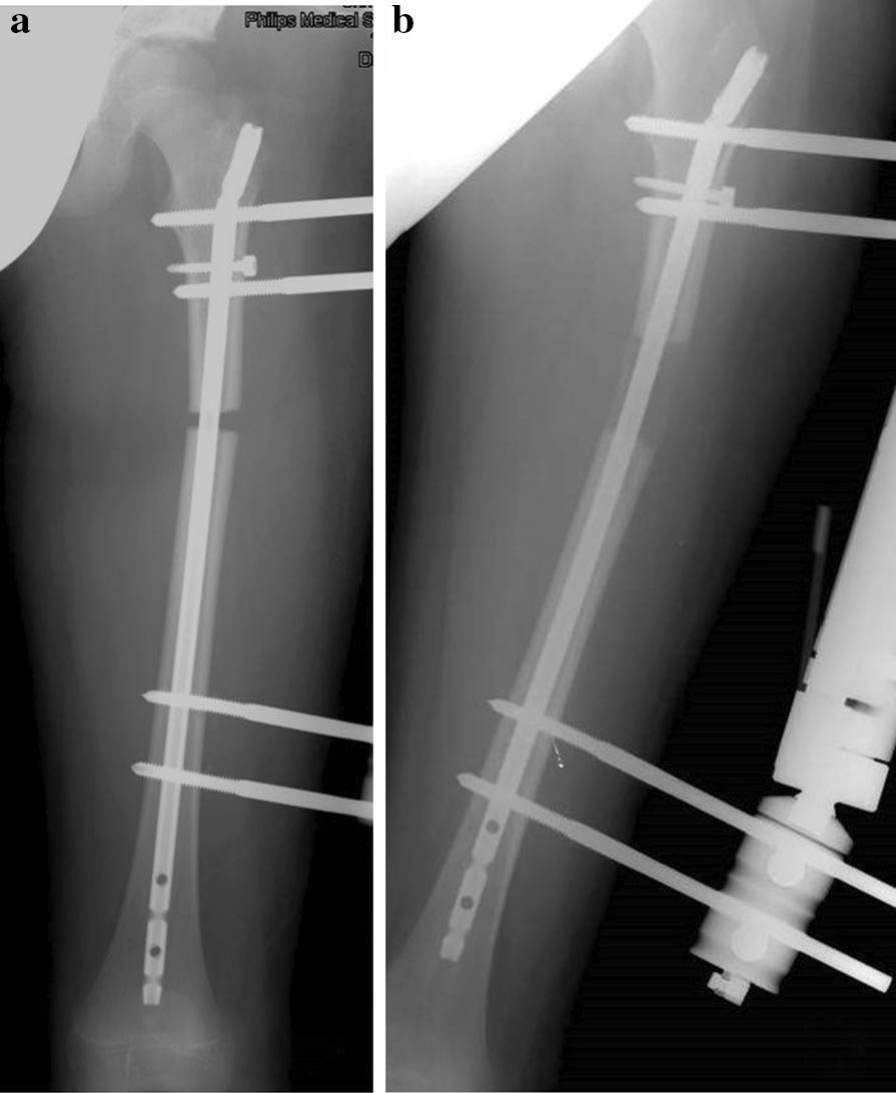
**a**, **b** The same patient as illustrated in Fig. 2. The left femur was elongated over an intramedullary nail according to Paley technique

**Fig. 4:**
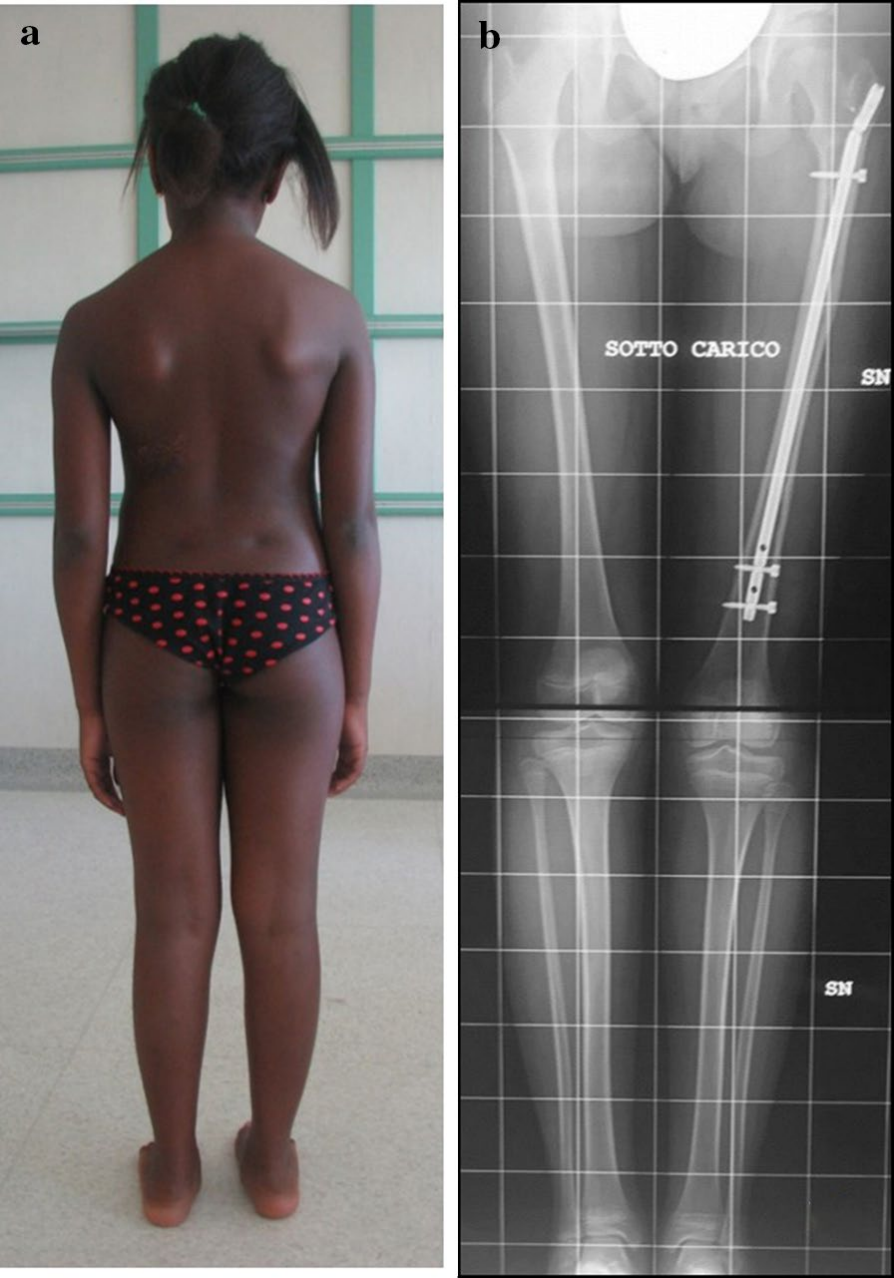
**a**, **b** The same patient as illustrated in Fig. 2, at bone healing. The patient showed excellent clinical correction of the deformity. Radiographically, the discrepancy of the femur was completely corrected, although a mild limb length discrepancy was still present, caused by shortening of the ipsilateral tibia

**Fig. 5:**
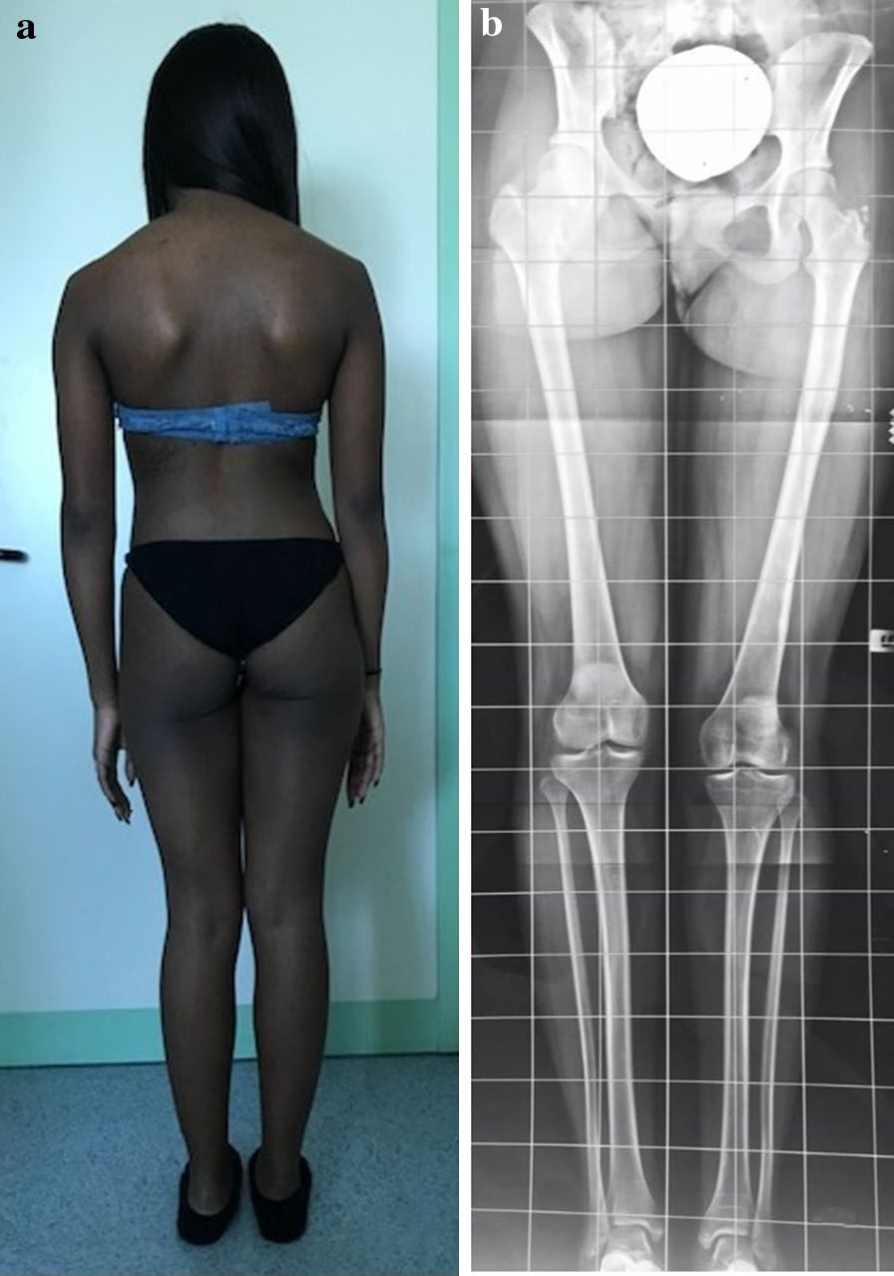
**a**, **b** The same patient as illustrated in Fig. 2. At follow-up, 10 years after lengthening, the clinical aspect of the girl is still excellent, without limping, in spite of a persistent radiographic limb length discrepancy due to 1.6 cm shortening of the tibia. An asymptomatic hypoplasia of the greater trochanter with associated bone fragmentation is present, caused by nail insertion during growth

Mean follow-up was 8 years (from 5 to 11 years). For femurs, the result was considered excellent in 4 cases, good in 12 cases (Figs. 2, 3, 4, and 5), fair in 3, and poor in 1 case. The patient with a poor result had a deep infection of the femur that forced us to remove the nail during lengthening, replace the external fixator, and administer antibiotic therapy for 4 months. At follow-up, the infection was eradicated, but the patient had a limb length discrepancy of 2.4 cm and a limp while walking, and was not satisfied with the final result. For tibiae, the result was considered excellent in four cases, good in three cases, and fair in one case.

We did not observe any statistically significant correlation between Paley score and patient age or lengthening achieved (Table 1). For femurs, the *r* values of the Pearson, Spearman, and Kendall correlations, between age and Paley score, were −0.394 (*p* value 0.085), −0.316 (*p* value 0.174), and −0.246 (*p* value 0.165), respectively, whereas those between lengthening and Paley score were −0.24 (*p*-value 0.307), −0.18 (*p*-value 0.442), and −0.145 (*p*-value 0.404), respectively. For tibiae, the *r* values of the Pearson, Spearman, and Kendall correlations, between age and Paley score, were 0.185 (*p* value 0.661), 0.207 (*p* value 0.622), and 0.154 (*p* value 0.610), respectively, whereas those between lengthening and Paley score were 0.573 (*p*-value 0.138), 0.524 (*p*-value 0.182), and 0.385 (*p*-value 0.202), respectively.

Five patients had superficial pin-site infection that resolved with a short course of antibiotic therapy, and one a deep infection (poor result). One patient had distal fiche breakage of the external fixator during femoral lengthening, which was removed and replaced. Another patient had breakage of both distal screws after removal of the external fixator, during the consolidation phase of the regenerated bone. The screws were removed using a lateral and medial approach. At follow-up, a 1.5 cm residual limb length discrepancy with mild axial deviation of the femur was present. Two patients showed a delay in consolidation of the regenerated bone, and one patient a complete lack of regenerate ossification of the tibia that required a new surgical operation. The patient was treated with application of a bone graft around the nail, taken from the iliac crest and the contralateral fibula. Despite this complication, at follow-up, we observed complete healing of the lengthened tibia, with a limb length discrepancy of 1.4 cm and no limp.

## Discussion

Bone lengthening for treatment of limb length discrepancy is a common surgical procedure performed using a circular or uniplanar external fixator. However, this technique may be complicated by axial deformities, fractures of the regenerated bone, and stiffness of adjacent joints [1–7]. To minimize these complications, Paley et al. [8] proposed performing femoral lengthening over an intramedullary nail and reported good results with the new technique in comparison with the Ilizarov lengthening method. From 1999 to 2011, other authors reported satisfactory results for femoral or tibial lengthening using Paley’s approach, albeit without control group comparison [9–12]. In these studies, the number of skeletal segments lengthened (femurs or tibiae) ranged from 9 to 118, the average age of the patients at surgery ranged from 16.2 to 26.6 years, the amount of lengthening ranged from 4.5 to 6.3 cm, while the rate of deep infection, the most feared complication with this method of treatment, ranged from 0% to 15%. We report herein excellent or good results in 82.1% of a series of patients with lower average age at surgery (14.2 years) compared with other studies, achieving similar average lengthening (5.5 cm) and a low deep infection rate (3.6%). This study, as well as those cited above, generally showed good results, without axial deviations during lengthening or fracture of regenerated bone after external fixator removal, and with the main advantage of reduced external fixation time. However, the major criticism of this technique is the elevated risk of infection, due to the presence of external fixator fiches that may transmit superficial infection along the intramedullary nail. To minimize this possible complication, some authors recommend avoiding any contact between the fiches and nail [13, 14]. We think that avoiding any contact between the fiches and nail is very challenging, although in some cases it can be achieved. However, we believe, in agreement with other authors [13], that in order to reduce the deep infection rate, it is fundamental to strictly supervise the patients during the external fixation time and immediately treat any pin-tract infection with antibiotics. We attribute our low infection rate to early and aggressive treatment of any pin-tract infection.

Several authors have reported that the consolidation time of the regenerated bone does not seem to be affected by the presence of an intramedullary nail [13, 16, 17]. On the contrary, other authors have reported a significantly longer consolidation time in the group lengthened over a nail [14]; however, the same authors justify this result by the presence in their series of two cases of deep infection, which were the real cause of the increase in the consolidation time of regenerated bone. In both of our groups, we observed in all only two cases of delayed consolidation, and only one patient required revision surgery, without an increase in the average consolidation time. However, we report significantly shorter consolidation time in tibiae compared with femurs. Another variable that influences the consolidation time, as well as the complication rate, is the lengthening percentage, which should not exceed 21.5% of the length of the skeletal segment involved [11]. In fact, some authors have reported delayed consolidation when the percentage of lengthening exceeded 33% [13]. In most of our patients, the lengthening did not exceed 20% of the length of the skeletal segment. The patients who had large lengthening did show a trend for worse results, but this difference was not statistically significant. This, however, could be due to the small number of patients who had large lengthening.

Some authors have described a variation of this technique called lengthening and then nailing, in which the intramedullary nail is inserted at the end of the lengthening phase [28]. The authors emphasized that this technique presents several advantages. It allows the use of a longer and thicker nail in order to better stabilize the regenerated bone; moreover, the reaming of the regenerated bone accelerates the consolidation process; finally, the technique avoids any contact between the fiches and nail, thereby reducing the risk of blockage of the bone sliding over the nail and the risk of deep infection. More recently, other authors reported a comparative study of tibial lengthening using this method compared with the traditional Ilizarov technique in two series of patients with short stature [29]. They observed decreased external fixation time and consolidation time in the patients treated with this method and recommended it in large tibial lengthening. These data were confirmed in a recent metaanalysis study [30]. We agree that this technique may be indicated in large tibial lengthening in which the circular external fixation controls possible axial deviations during lengthening, but it is not recommendable in femoral lengthening in which a uniplanar external fixator is used and controlling axial deviations is more difficult.

Some authors suggest using an unreamed nail, as they believe that reaming the intramedullary canal might compromise the endosteal blood supply [13]. On the contrary, we believe, in agreement with other authors [14, 15], that reaming has the advantage of obtaining a larger canal in order to facilitate bone sliding on the intramedullary nail, without any consequence for healing of the regenerated bone. Another possible complication related to reaming is the increased risk of fat embolism; in our series, in which reaming was performed in all cases, we did not observe any fat embolism.

Recently, intramedullary lengthening nails have been developed, such as the mechanical Intramedullary Skeletal Kinetic Distractor and the more recent motorized nails such as the Fitbone and Precice [18–27]. Encouraging results have been reported with these devices, with various authors describing satisfactory outcomes in femoral and tibial lengthening, without the discomfort caused by the external fixator. However, as reported by some authors [15, 18, 25], these devices are still very expensive and may cause frequent complications, such as abnormal distraction length, nail breakage, malfunctioning external remote controllers, and pain. Therefore, they still require longer follow-up studies for definitive evaluation.

The limitations of this study are its retrospective nature and the lack of a control group.

In conclusion, we believe, on the basis of our results, that lengthening over an intramedullary nail still represents a valid option for treatment of lower limb discrepancy, because it reduces the external fixator time and the risk of axial deviations, fractures of the regenerated bone, and joint stiffness. Particular attention should be paid to the risk of deep infection by means of early and aggressive treatment of any pin-tract infection.

## Data Availability

Not applicable.
